# An Improved Partial Discharge Detection System Based on UV Pulses Detection

**DOI:** 10.3390/s20174767

**Published:** 2020-08-24

**Authors:** Zeliang Shen, Jingang Wang, Gang Wei

**Affiliations:** 1 School of Electrical Engineering, Chongqing University, Chongqing 400044, China; jingang@cqu.edu.cn; 2 School of Electrical Engineering, Chongqing University of Science and Technology, Chongqing 401331, China; 2017012@cqust.edu.cn

**Keywords:** partial discharge (PD), UV pulses, UVTRON sensor, detection system

## Abstract

Partial discharge (PD) usually reflects failures and potential hazards of equipment, so PD detection is important to protect the power system. The most reliable method now is the pulse current method (PCM), but the device of PCM is large and hard to carry. Ultraviolet (UV) pulse detection is another method to detect PD, which has a high precision, strong anti-interference ability, and a long effective distance. However, the existing detection system does not work well when the PD is weak and can hardly reflect the hidden trouble of equipment. This paper introduces an improved PD detection system, based on the UV Pulse Method, which is of high precision and can reflect early discharge. In this study, a corresponding detection device was also built. This device is handheld, non-contact, easy to use, and of high precision.

## 1. Introduction

Partial discharge (PD) accelerates the deterioration of equipment insulation and causes the failure of electric equipment [1,2,3]. As insulation failures account for approximately 80% of total failures, PD detection has become the most important way to spot equipment failures and potential hazards [4]. Pulse Current Method (PCM) is the most commonly used method of PD detection and have the highest sensitivity and accuracy for detecting PD inside the insulation. However, for PCM, the process is complicated and the anti-interference ability is weak, which makes routine inspections difficult. UV can be generated during partial discharges. In recent years, UV Imaging Method (UVIM) was widely used for PD detection, and existing research studies proved the mathematical relationship between the discharge voltage and the detection results [5]. Compared to PCM, UVIM has advantages of high sensitivity, non-contact, anti-interference ability, and a high positioning accuracy [6,7,8]. However, the corresponding device is big, expensive, and complicated to operate [9,10]. In addition, it is difficult for UVIM to detect early danger when PD is weak. UV Pulse Detection (UVPD) is another way to find PD through UV light [11,12,13]. UVPD uses a UV sensor to detect UV signal and translates it into waveforms through the circuit. The parameters of the waveform reflect the strength of the PD. The UVPD method is sensitive and can response to weak UV signal [14,15]. In addition, the device is light and cheap, which can be used in routine inspections of equipment [16,17]. However, existing UVPD devices have a simple circuit design, and have little idea of parameter optimization [18,19,20]. This makes the detection results unstable and decreases the accuracy. When the PD is weak, it would be difficult for the UVPD to detect the signal, thus the detection system must be highly sensitive [21,22,23,24]. Through circuit improvement and parameter optimization, the portability and sensitivity of UVPD device can be improved, and it meets the needs of routine inspections.

This study introduces a PD detection system based on UVPD. The system consists of a solar-blind UV sensor and processing circuit. The circuit was redesigned and the parameters are improved to make the system sensitive enough to detect early PD. A handheld, non-contact, and easy to use device was built based on this system, which could detect PD in a convenient and flexible way. A test platform was set up to test the accuracy of the device under various environment, and data were compared with that obtained by PCM. Test results showed that the device was sensitive enough and could be used for early PD detection.

## 2. Circuit Optimization of the UV Pulse Detection System

### 2.1. Principle of the UV Pulse Method

Most of the UV light generated by a PD is in the range of 280–400 nm [25]. While a small part of the UV light is in the range of 160–280 nm, which is usually called the solar-blind UV light. This kind of UV light can be used as the detection target, since it can be hardly interfered by sunlight. We used a solar-blind UV photodiode (UVTRON), with a working wavelength range of 160–280 nm, to collect the UV signal, so that the interference of the visible light could be removed.

When PD occurs, UV radiation is generated and the strength increases with increase in PD strength [26]. The discharge signal of the electric device can turn into voltage pulses through a UV light-sensitive tube. The number of pulses generated by the detection system can reflect the intensity of the discharge. The number of pulses collected per unit time is positively related to the discharge intensity.

### 2.2. Detection System

**Figure 1:**
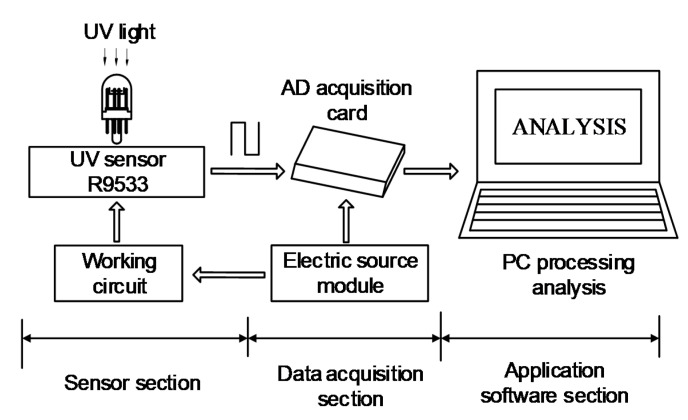
Block diagram of the UV Pulse Detection System hardware.

As shown in Figure 1, the UV pulse detection system consists of two parts—the hardware circuit and the PC-side software program. The detection system uses a solar-blind UVTRON sensor as the detection sensor, and collects data through a data acquisition card. Then, the data are analyzed and the pulse waveform is displayed on the PC.

When the system works, the UVTRON receives the solar-blind UV light, and when the intensity of the UV light reaches a certain level, the UVTRON turns on. The driving circuit converts the continuous light signal into the continuous pulse voltage signal. The data acquisition card directly collects the output pulse voltage signal, transmits it to the PC side for analysis, and processing occurs. Finally, the waveform and parameters of the pulse is showed to determine the discharge of the equipment.

### 2.3. UVTRON Sensor

Existing research studies usually use UVTRON-R2868 as the sensor of UV pulse detection system [3,23,26,27]. R2868 is a side window type UVTRON, which has a sensitivity of 5000 cpm (the cpm is defined as the number of detected pulse per minute under UV of 200 nm wavelength and 10 pW/cm^2^ radiation degree). In addition, there is another kind of UVTRON called the R9533. The R9533 is an end-window type UVTRON with a sensitivity of 10,000 cpm, which is more sensitive than the R2868. Additionally, the end-window type sensor has advantages of convenient installation and easy alignment of the measuring point. There is translucent deposits on the inner surface of the entrance window of the R9533, which allows the R9533 to have a better uniformity than the side-window type sensors, and finally increases the UV flux [25,28].

Integrating the process cost, working stability, sensitivity, and some other factors of the existing UV sensors [29,30], the type R9533 sensor made by HAMAMATSU Corporation from Japan, was found to have the best performance and was selected as the UV pulse detection sensor.

### 2.4. Drive Circuit Principle

#### 2.4.1. Drive Circuit Principle

**Figure 2:**
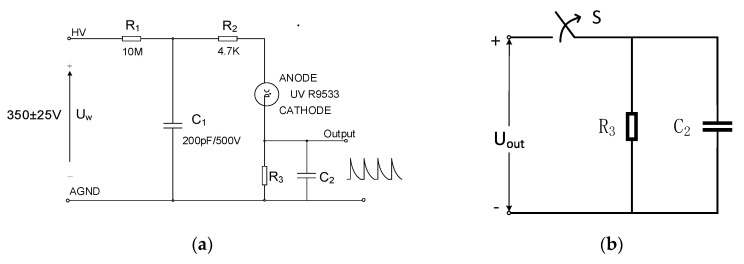
UVTRON drive circuit. (**a**) Recommended operating circuit. (**b**) The equivalent circuit on the output side.

Existing studies [3,11] usually use the drive circuit [25] recommended by the HAMAMATSU corporation, as is shown in Figure 2a. According to the user’s manual [28], a 350 ± 25 V DC power supply is required to be the source of the circuit. When the UVTRON is on, the photocurrent is generated and the pulse voltage is detected on the detection resister R_3_. This circuit is usually used for flame detection [26].

**Figure 3:**
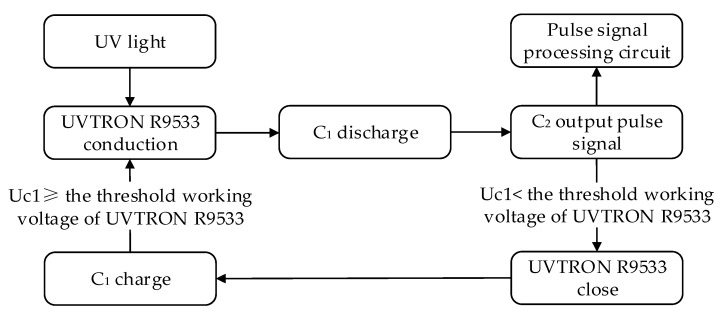
Drive circuit block diagram.

Figure 2a shows that the circuit is a typical first-order circuit; when the circuit starts working without a UV incident, the R9533 is off. When the photocathode receives the incident UV light, the R9533 turns on. Then, the potential of R3 rises and the voltage between the two poles of the R9533 drops. The electric field weakens until the R9533 turns off. The block diagram is shown in Figure 3.

When there is a continuous UV light incidence, the UVTRON-R9533 drive circuit generates the continuous output voltage pulse. When the UV sensor is on, the C_2_ charges; and when the sensor is off, an RC loop forms at the output end of the circuit. The parameters of the RC loop affect the characters of the output pulse. The width of the output pulse is determined by the time constants of this circuit. In addition, the on time of the sensor is directly related with the detection resistor R_3_; if R_3_ is larger, the bipolar voltage of the R9533 drops faster, thus, the on time is shorter; and conversely, if R3 is smaller, the conduction time is longer. Based on experimental tests, the on-time changes within approximately 1 µs, which can be seen as unchangeable; thus, the R9533 is equivalent to a switch. The equivalent circuit of the output part is shown in Figure 2b.

#### 2.4.2. Drive Circuit Design

**Figure 4:**
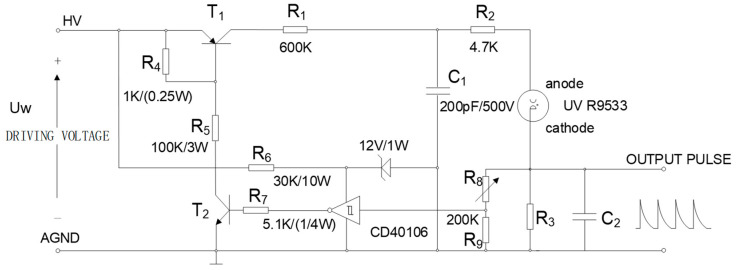
Optimized UVTRON driver circuit; (I_R8_ + I_R9_) << I_R3._

Since the resistance R_1_ of the driver circuit is large, the voltage on the capacitor C_1_ recovers relatively slowly, which would reduce the detection frequency. Thus, we redesigned the drive circuit to improve the sensitivity of the UVTRON, as is shown in Figure 4.

In the initial state, the transistor T_1_ and T_2_ are open. When the UV light enter the UV sensor, the circuit generates an output pulse, and the rising edge of the pulse is detected by the CD40106. Then, the T_1_ and T_2_ closes. The voltage on R_3_ quickly rises until the discharge of the sensor disappears, then the circuit is back to the initial state. The new circuit reduces the resistance value of R_1_, shortens the charging time of capacitor C_1_, and the arcing time of the sensor, which improves the sensitivity and the detection frequency of the UV pulse detection system. In addition, the values of R_3_ and C_2_ are defined in Section 2.4.3.

#### 2.4.3. Parameter Optimization

When the power supply U_w_ changes, the electric field intensity between poles of the UV sensor changes. When the U_w_ increases within a certain range, UV light is easier to cause ionization and thus to cause discharge. Then, the photocurrent increases and the amplitude of the output pulse also increases. So, it is necessary to increase U_w_ to detect weak corona discharge signal. However, when U_w_ increases too much, it is easy for the sensor to form self-sustained discharge, which reduces the accuracy of the UV sensor. Therefore, it is important to decide the appropriate value of U_w_.

The amplitude of the output pulse is determined jointly by the power supply U_w_, the resistance R_3_ and the capacitance C_2_, and the width of the pulse is determined by the RC loop consisting of R_3_ and C_2_. Thus, we improve the performance of the circuit by changing the value of U_w_, R_3_, and C_2_.

(1) Determining the Drive Voltage U_w_

**Figure 5:**
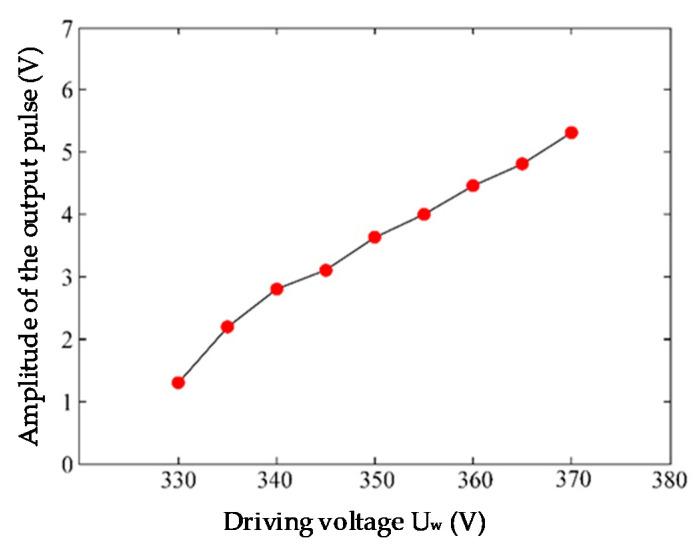
Output pulse amplitude under different drive voltages (R_3_ = 10 kΩ, C_2_ = 1000 pF).

According to the recommended drive circuit parameters [28], R_3_ = 10 kΩ and C_2_ = 1000 pF. The driving voltage U_w_ is limited within 325~375 V, according to the rated working voltage of the R9533 sensor. A 40 Hz spark generator is used as the discharge source. The amplitude of the output pulse changes as the U_w_ changes. The relationship between the driving voltage and the pulse amplitude is shown in Figure 5.

As the driving voltage increases, the amplitude of the pulse output increases, which ranges from 1 to 7 V. When the sensor is on, the current should not exceed 1 mA, which is the average discharging current, to make sure the circuit works steadily.

**Figure 6:**
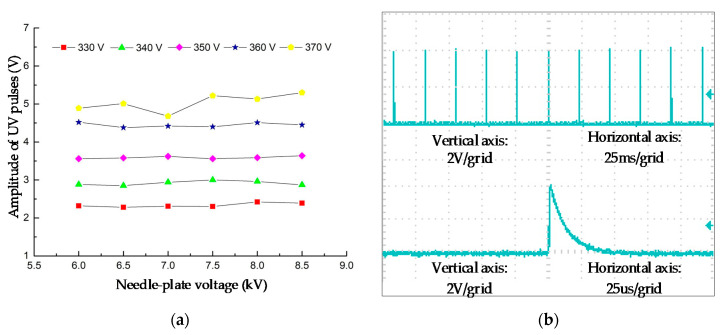
UV pulse amplitude and waveform after parameter optimization. (**a**) UV pulse amplitude under various driving voltages. (**b**) UV pulse voltage waveform.

Figure 6 shows how the UV pulse amplitude changes, when the needle-plate voltage and the driving voltage changes. As can be seen from Figure 6a, the amplitude of the output pulse increases when the driving voltage increases. Moreover, when the driving voltage is 350 V, the amplitude of the output pulses has the least change when the needle-plate voltage changes. In addition, Figure 6b shows the waveform of UV pulses when the driving voltage and needle plate voltage are 350 V and 7.5 kV, respectively.

Therefore, the driving voltage U_w_ should be set as 350 V. Further experiments showed that the best value of U_w_ does not change when the R_3_ and C_2_ changes.

(2) Determining R_3_ and C_2_

**Figure 7:**
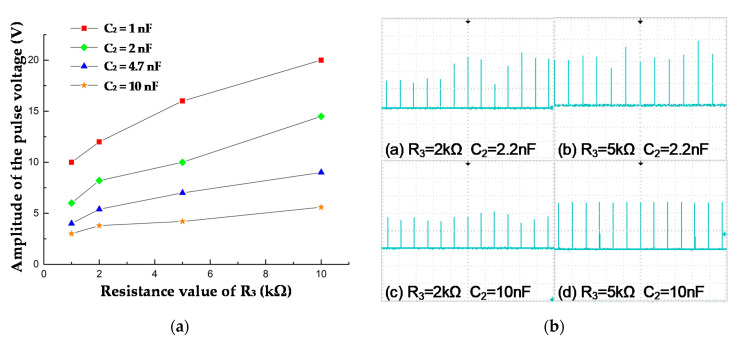
The output pulses under different RC parameters. U_w_ = 350 V. (**a**) Impulse voltage amplitude under different RC parameters. (**b**) Impulse waveform under different RC parameters.

Set the value of U_w_ as 350 V. Change the value of R_3_ and C_2_, and then observe the amplitude and width of the output pulse. Figure 7 shows how the UV pulse amplitude changes when C_2_ and R_3_ changes.

As seen from Figure 7a, when the resistance R_3_ is unchanged, the amplitude of output pulses increases when the capacitance C_2_ decreases; when the capacitance C_2_ is unchanged, the amplitude increases when the resistance R_3_ increases. Figure 7b shows the waveform of UV pulse, under different RC parameters. When R_3_ = 5 kΩ and C_2_ = 10 nF, the amplitude of pulse changes the least, which indicates that the change of the UV sensor discharge current is minimal, and the capacitor can best set the output voltage. At this time, the voltage amplitude is 4.16~4.28 V. Figure 6b shows different waveforms of output pulses under different values of R_3_ and C_2_. It can be seen that the waveform is very smooth, under the condition when R_3_ = 5 kΩ and C_2_ = 10 nF.

In summary, when U_w_ = 350 V, R_3_ = 5 kΩ and C_2_ = 10 nF, the output waveforms have the smallest amplitude fluctuation, which is beneficial to the statistics of the data.

### 2.5. System Test

**Figure 8:**
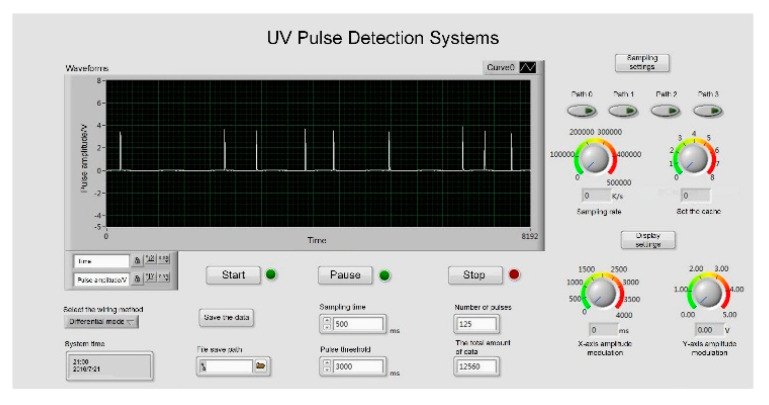
UV discharge detection system test.

It was necessary to test the detection performance of the system. We used LabVIEW to collect and analyze the data of the output pulses, as is shown in Figure 8. The waveform was clear and matched that detected by the oscilloscope, which indicated that the detection result was accurate.

**Figure 9:**
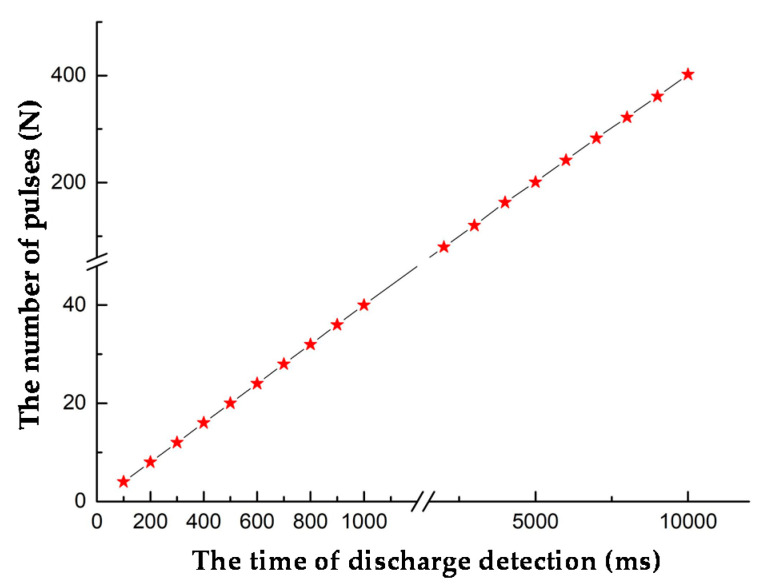
Test results of the periodic UV discharge system.

The number of output pulses between 100 to 10,000 ms was counted. As shown in Figure 9, the number of output pulses was linearly related to time, indicating that the frequency fluctuation level was low, which proved that the system could accurately detect the discharge pulse.

## 3. Evaluation

A number of practical experiments were carried out to evaluate the performance of the system. The system worked under various influence factors to analyze the dynamic range. Finally, the detection sensitivity of the UV pulses method and the pulse current method was compared.

### 3.1. Test Platform

**Figure 10:**
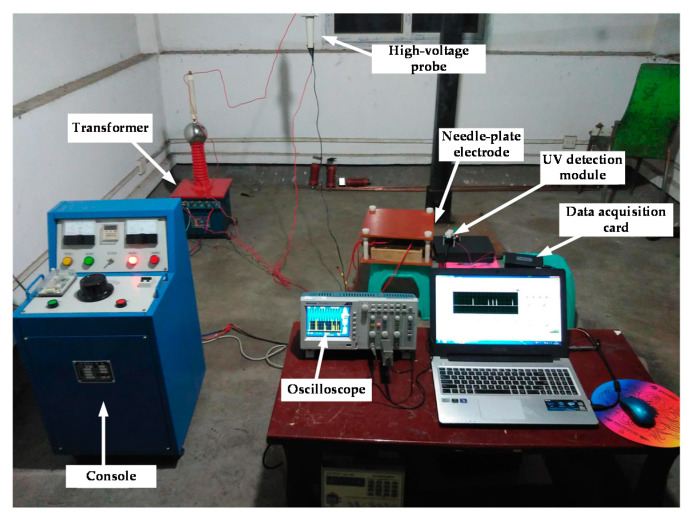
Experimental platform.

To ensure that the detection of a corona discharge of the electrical equipment was under high-voltage, the experiment platform, as shown in Figure 10 was established. According to the laboratory measurement, the voltage of the experimental AC source was between 0~20 kV.

**Figure 11:**
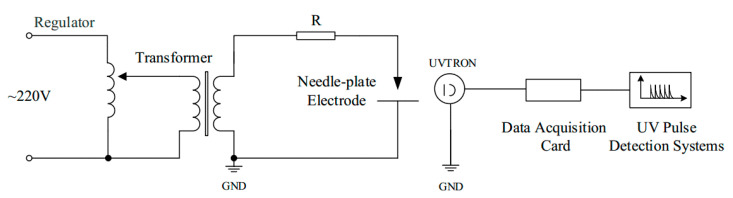
Needle-plate corona discharge test circuit.

As shown in Figure 11, a test circuit for a corona discharge experiment was prepared. The needle-plate electrode was made of metal. The tip of the needle electrode was processed into a spherical shape with a diameter of approximately 0.5 mm. The plate electrode was processed into a steel plate with a diameter of 5 cm and a smooth surface without burrs. The plate electrode gap distance could be adjusted from 1 cm to 5 cm. During the corona discharge test, the UV pulse detection system was placed in the needle-plate discharge direction, to ensure that the UV detection sensor could receive the strongest corona discharge UV signal, reflecting the discharge situation.

### 3.2. Sensitivity Test under Different Factors

In the corona discharge experiment, factors such as voltage (U), detection distance (L), and distance between the needles and plates (D) of the needle-plate electrode all affected the number of UV pulses (N) produced by the UVTRON. Therefore, these influencing factors were taken into consideration to carry out relevant experiments.

#### 3.2.1. Sensitivity Test under Different Needle-Plate Voltages

Set the needle-plate gap distance D = 1 cm, the detection distance L = 10 cm, and the needle-plate voltage to be from 4 to 12 kV (gap breakdown occurred when the voltage was approximate 13 kV). The number of UV pulses was counted.

**Figure 12:**
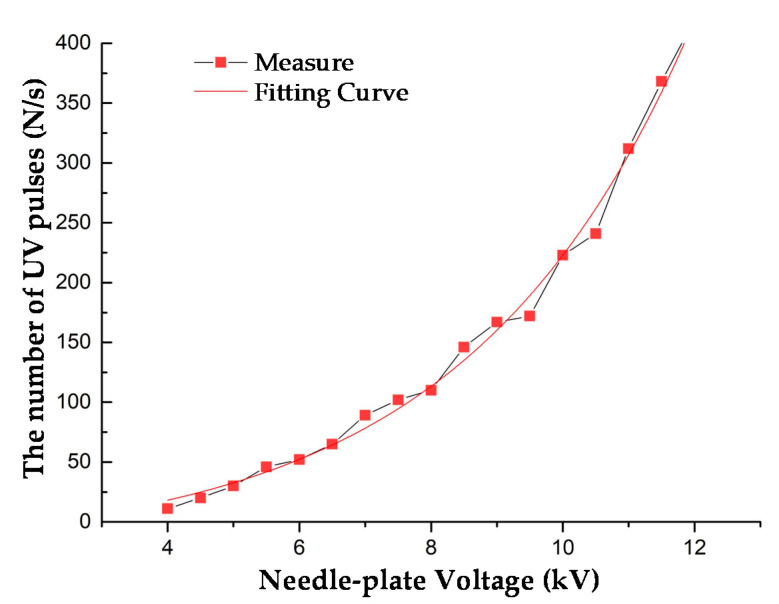
Relationship between needle-plate voltage and the number of UV pulses (D = 1 cm, L = 10 cm).

The relationship between the needle-plate voltage and the number of UV pulses is shown in Figure 12. The result showed that the number of UV pulses was positively related to the needle-plate voltage. When the voltage was lower, the number of UV pulses increased slowly; and when the voltage was higher, the number of pulses increased rapidly. Based on engineering experience, the AC voltage and the number of UV pulses satisfied the exponential function. The fitting formula was:

(1) $$N=0.2709U^{3.0499}$$

where *N* is the number of UV pulses and *U* is the needle-plate voltage. In addition, the fitting degree R_2_ was 0.9833.

#### 3.2.2. Sensitivity Test under Various Detection Distances

Setting the needle-plate gap distance D = 3 cm and the needle-plate voltage U = 12 kV. This made the corona discharge intensity to be relatively high and thus made it easier to determine the relation between the detection distances and the number of UV pulses.

**Figure 13:**
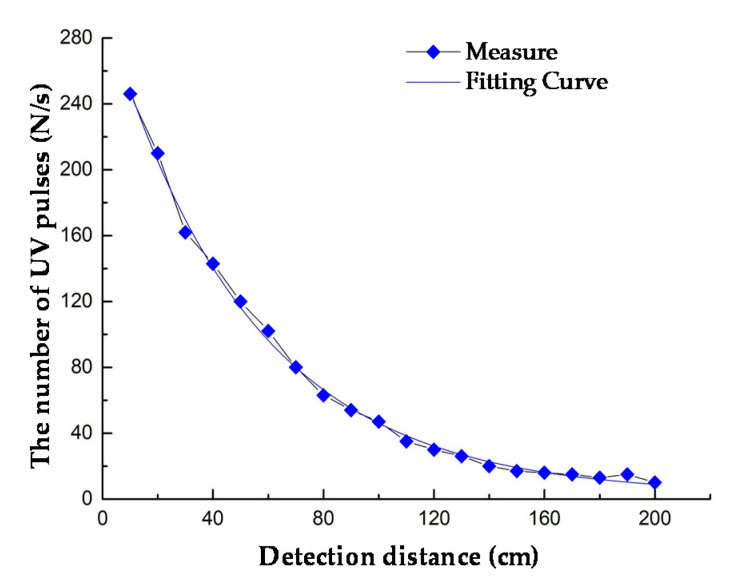
Detection distance and the number of UV pulses (D = 3 cm, U = 12 kV).

The relationship between the detection distance and the number of UV pulses can be seen in Figure 13; as the detection distance increased, the number of UV pulses gradually decreased. Based on engineering experience, the detection distance and the number of UV pulses satisfied the power function. The fitting formula was:

(2) $$N=8433.8L^{-1.188}$$

where *N* is the number of UV pulses and *L* is the detection distance. In addition, the fitting degree was 0.9907.

#### 3.2.3. Sensitivity Test with Different Needle-Plate Gap Distance

Set gap distance D = 1 cm, 3 cm, and 5 cm, and the detection distance L = 10 cm. When the voltage between the needle and plate increased, the electric field strength at the tip of the needle gradually increased, and the intensity of the UV light received by the UVTRON was greater, resulting in a greater frequency of UV pulses.

**Figure 14:**
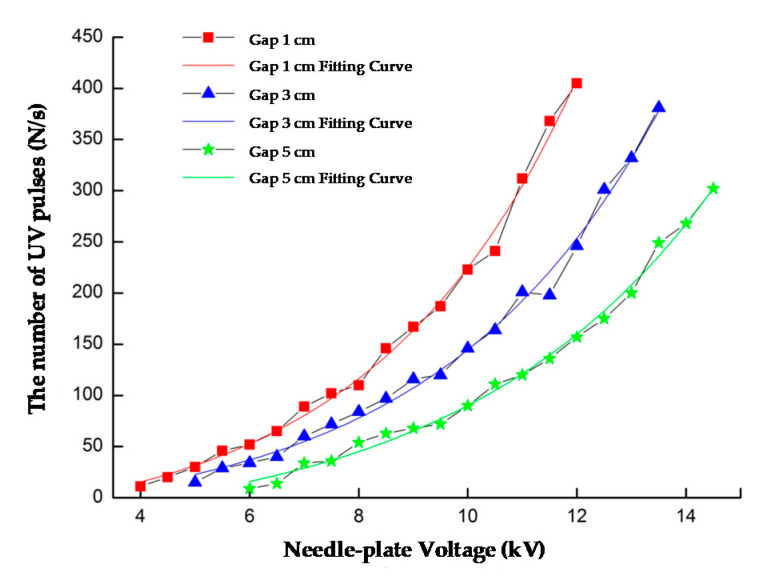
Needle-plate gap distance and the relationship between the number of UV pulses (L = 10 cm).

The fitting curve is shown in Figure 14, and the fitting formulas are shown in Table 1. The initial discharge voltages and the corresponding UV pulses changed, when the gap distance changes. When the gap voltage remains unchanged, the discharge intensity decreased as the gap distance increased.

The test results showed that the number of detected UV pulses had a good fitting relationship with needle-plate voltages, the detection distance and the needle-plate gap distance. In addition, the fitting degree R2 was above 0.98, indicating that the UV discharge detection system had a high detection accuracy.

### 3.3. Analysis of Dynamic Range

We analyzed the dynamic range of the detection system to make sure the detection result under different PD situations was reliable. A number of different needle-plates was used to conduct the experiments.

**Figure 15:**
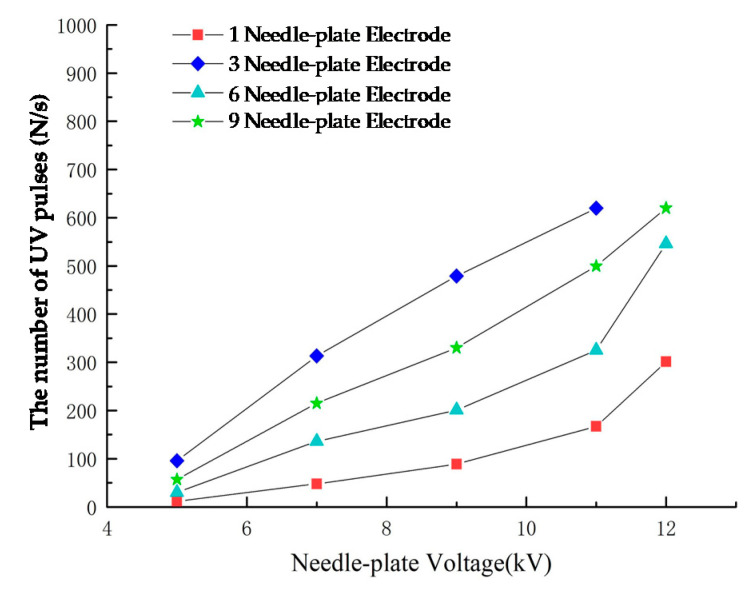
The relationship between the number of needle plates and the number of UV pulses (D = 3 cm, L = 15 cm).

**Figure 16:**
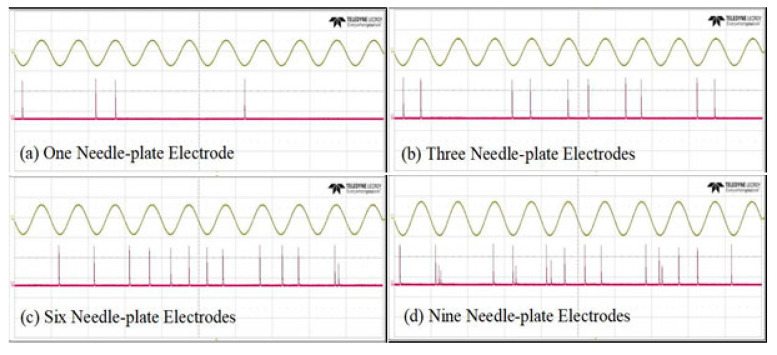
UV pulses under different numbers of needle-plates (D = 3 cm, L = 15 cm, U = 5 kV).

When the cathode of the UVTRON faced the discharge source, the sensor received the largest amount of UV light and had the highest sensitivity. Thus, the needle-plate models were arranged in a horizontal line. The distance between adjacent tips was 5 cm, the needle-plate gap distance was 3 cm, and the detection distance was 15 cm. The voltage of the needle-plate rose from 0 kV to 12 kV. The number of UV pulses and the resolution of the discharge detection in 1 s was observed. The test results are shown in Figure 15 and Figure 16.

(1) As seen in Figure 15, when there were 1 or 3 gaps discharging, the number of UV pulses rose exponentially as the gap voltage increased. When there were 6 or 9 discharging gaps, the waveform was very dense, and the number of pluses increased at a slower rate, as the gap voltage increased. In addition, the system always had a high resolution in both situations. The proper reason was that when the discharge was strong, the detection system could not detect all pulses due to the limit of the UVTRON.
(2) The needle-plate voltage was set at U = 5 kV. When there was only one discharging gap, the system produced less pulse signal. As the number of needle-plates increased, the number of UV pulses increased significantly, indicating that UV pulse detection system could be applied to early detection of PDs.

### 3.4. Comparison with the Pulse Current Method

To verify the reliability of the UV discharge detection system, we carried a series of experiments to compare the detection results between the UV discharge system and the pulse current method. The test room was nearly under the standard atmosphere pressure and the relative humidity was about 60% to 80%.

The pulse current method used the initial discharge voltage Ui, the apparent charge q, the average discharge current I, and other parameters to measure the discharge intensity, and further reflect the insulation condition. The main drawback was that the detection result could be easily disturbed by the surroundings.

In this research, we used a PD-detection device with model HZJF-123. We used standard wiring (device and discharge source in parallel) to make the test results stable. Voltage was applied across the sample and results were obtained in the device. Then, the results were compared with those obtained by the UV pulse method.

#### 3.4.1. Comparison on Initial Discharge Voltage U_i_

The initial discharge voltage U_i_ was easy to measure. Therefore, we set the needle-plate device to compare the initial discharge voltage of the two systems.

The test distance was set at L = 15 cm, and the needle-plate gap distances were D = 1 cm, 3 cm, and 5 cm, which characterized the intensity of the corona discharge. The test results are shown in Table 2.

The gap distance was set to be 1 cm. The initial discharge voltage for the UV pulse detection was 3.8 kV, and the initial discharge voltage for the pulse current method was 5 kV. In addition, Table 2 shows that when the needle-plate gap distance was the same, the initial discharge voltage detected by the UV pulse detection system was 25% lower than that detected by the pulse current method. Moreover, as the needle-plate gap distance increased, the difference increased.

#### 3.4.2. Comparison on the Number of Detected Pulse

**Figure 17:**
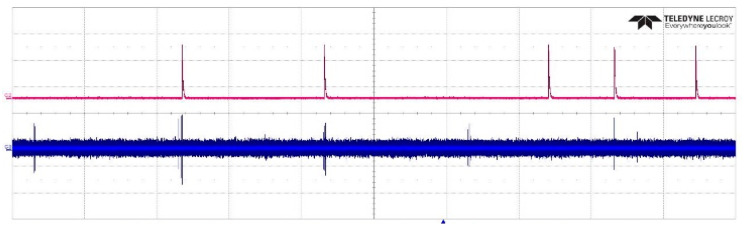
Phase contrast of a UV pulse and the corona current pulse.

In the experiment, the distance between the needle-plate gap was D = 1 cm, the detection distance was L = 15 cm, and the needle-plate gap voltages U were 4, 5, 6, 7, 8, and 9 kV. The number of output pulses of the UV pulse detection system and the pulse current method system in 200 ms, was counted. The experimental results are shown in Table 3 and Figure 17.

With an increase in the discharge intensity, the number of output pluses of both systems increased significantly, and the pulse number of the pulse current method increased faster than that of the UV pulses detection system.

When the gap voltage was 4 kV, the number of output pulses detected by UV pulses detection system was 5 and that detected by the pulse current method was 0, which proved that the UV pulse detection system could detect the weak corona discharge. As shown in Figure 17, the phase of the UV pulse was consistent with the phase of the corona current pulse. The corona current pulse had both positive and negative pulses and had a low amplitude, which made it easy to be influenced by interference. The amplitude of the UV pulse was approximately 4 V and was more insensitive to the noise, which made the measurement results more accurate.

#### 3.4.3. Apparent Charge Amount of the Circuit

The pulse current method could be used to quantitatively describe the discharge of a circuit. The needle-plate D = 1 cm and the detection distance L = 15 cm was set (which is the same as Section 3.4.2). The needle-plate voltage was set and the apparent charge amount measured by the pulse current method was recorded. The result is shown in Table 4.

The above tests showed that the UV discharge detection system was reliable and could be used for early detection of PD.

### 3.5. Corresponding Device

**Figure 18:**
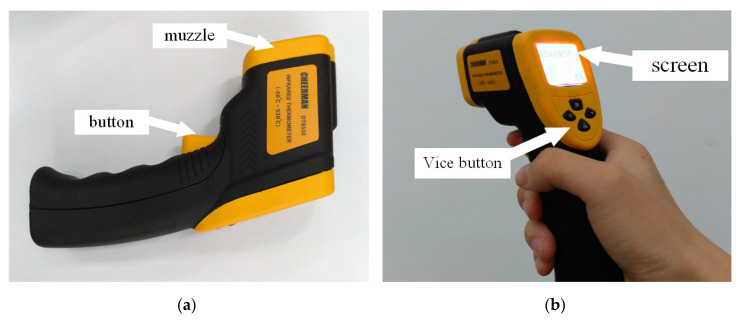
The PD detection device based on the UV Pulse Detection. (**a**) Main view. (**b**) Side view.

Based on the improved system, we build the corresponding device to provide a non-contact, convenient, and flexible method to realize early PD detection. The device was handheld and easy to use. The muzzle was pointed at the place to be tested and the button was pushed; the test result appeared in seconds. The photo of the device is show in Figure 18.

## 4. Conclusions

This study introduced a PD detection system based on UV Pulse Detection. The system was of high precision and could reflect early PD, and the process circuit was improved to provide better performance. The corresponding device was handheld, non-contact, easy to use, and could detect PD in a convenient and flexible way. The experiment platform was set up to verify the performance, and the results were as follows:

(1) Compared with the conventional circuit, the pulse width was about 42 µs, while the pulse width of the conventional circuit was about 175 µs. The accuracy and sensitivity was better.
(2) The pulse number detected by the UV pulse detection system had a functional relationship with the needle-plate voltage and the detection distance. The experimental data and the fitting formulas had a good fitting degree, which was above 0.98.
(3) As the intensity of the discharge increased, the number of UV pulses measured per unit time increased significantly. When the discharge was stronger, the increase rate of the pulse number began to decrease, which was due to the limitation of the UVTRON.
(4) The UV pulse detection system had flexibility, a non-contact characteristic, and an anti-interference ability, and it could provide a cost-friendly plan for real-time monitoring of the equipment. The test results of the pulse current method showed that the UV method was sensitive enough and could be used in early detection of PD.

It is worth mentioning that the test results were obtained in laboratory, based on the needle-plate discharge model. More experimental studies are still needed in future research.

## Figures and Tables

**Table 1:** Fitting of different needle-plate gap distance and UV pulse number.

D/cm	Fitting Expression	Fitting Degree R2
1	$N=0.2079U^{3.0499}$	0.9907
3	$N=0.1654U^{2.959}$	0.9887
5	$N=0.0235U^{3.5736}$	0.9912

**Table 2:** UV pulse detection and pulse current method compared to the initial discharge voltage U_i._

D/cm	UV Pulse Detection/kV	Pulse Current Method/kV
1	3.8	5
3	4.9	6.8
5	6.0	8.2

**Table 3:** Detection pulse number of UV detection and the pulse current method.

U/kV	UV Pulse Number	Corona Current Pulse Number
4	5	0
5	9	4
6	18	68
7	56	156
8	103	353
9	125	429

**Table 4:** Detection pulse number of UV detection and the apparent charge amount of the circuit.

U/kV	UV Pulse Number	Apparent Charge Amount/pC
5	9	200
7	56	400
9	125	500
